# The Evaluation of the Vector System in Removal of Carious Tissue

**DOI:** 10.1155/2010/821357

**Published:** 2010-04-29

**Authors:** Mine Yildirim, Figen Seymen, Nurullah Keklikoglu

**Affiliations:** ^1^Department of Pediatric Dentistry, Faculty of Dentistry, Istanbul University, Istanbul 34093, Turkey; ^2^Department of Histology and Embryology, Faculty of Dentistry, Istanbul University, Istanbul 34093, Turkey

## Abstract

The aim of this study was to evaluate the Vector system in comparison to the conventional technique in cavity preparation. Four extracted primary teeth with no restorations and similar fissure carious lesions and four permanent teeth extracted for orthodontic reasons were used. Class I preparations were made provided that the caries depth remained within the dentin limits. Two teeth were treated with an aerator, the other two had carious tissue removed with the Vector system. Prepared cavities were evaluated with scanning electron microscopy for the surface roughness of the dentine and enamel and for the carious tissue removal efficiency. This pilot study determined that it is possible to remove carious tissue and perform cavity preparation with the Vector system. According to this preliminary evaluation of surface quality, a cavity prepared with the Vector treatment system, allows for a slicker floor, and a more regular enamel-dentine line than that prepared with an aerator. However, the Vector system requires a longer treatment time which we believe may be a negative point, especially for young patients.

## 1. Introduction

Conventional cavity preparation and carious tissue removal are based on Black's principle of extension for prevention. This principle requires removing healthy tooth structure which is very destructive and leads to excessive tissue loss. In recent years, minimal invasive cavity preparation has gained popularity. Current practice keeps the size of cavities as small as possible. Conservative cavity preparation, which includes handpieces and burs, leads to the undesirable removal of healthy tooth structure. Due to this excessive loss of sound tissue, efforts have focused on new techniques [1, 2]. 

Over the last few years, new techniques and procedures for hard tissue removal were developed as alternatives to the conventional mechanical procedure [3]. Alternative carious dentin removal techniques have been proposed, including hand excavation, air-abrasion, air-polishing, ultrasonication, sonoabrasion, lasers, and chemomechanical methods [4, 5] (Table 1).

The Vector system is a new method combining both ultrasonic effects and microabrasive action of quartz crystal suspension. This method uses specially shaped metal tools for use with an abrasive slurry of silicon carbide (Vector Fluid Abrasive, grain size 40–50 mm) for micro-invasive preparation countouring and finishing of the tooth substance and nonmetal restorations. These instruments are available in different cylindrical and oval shapes for a precise preparation. The tissue removal is accurate, athermal, and of a gentle nature. Heat induction is almost eliminated so that only a small amount of liquid is needed. As a result the conditions of work during the preparation of carious lesions in teeth remain unchanged [6].

The manufacturer suggests that the Vector removes biofilm, plaque, calculus, and endotoxins. The resonating ring of the Vector System converts the ultrasonic dynamics of 25 kHz in a similar way to a hula-hoop. If it is pressed into the horizontal position it moves vertically with 90 degrees deflection. This allows a linear movement of the instruments parallel to the tooth surface and an adhering film of water or particle suspension. The ultrasound's energy is thereby indirectly coupled onto the tissues to be treated.

In contrast to diamond instruments, in the Vector system the energy is transmitted indirectly via the silicon carbide particles (average grain size approximately is 50 *μ*m) carried in the water. Preparation with the Vector system is therefore carried out without exposure to high temperatures [7]. 

Therefore, the purpose of this study was to evaluate the Vector system in comparison to the conventional air-motor technique.

## 2. Materials-Methods

Four extracted primary teeth without restorations and with similar fissure carious lesions, and four permanent teeth extracted for orthodontic reasons were used. Class I cavities were prepared, provided that the cavity depth remained within the dentin limits. Two primary and two permanent teeth were treated with an aerotor, and in the other group teeth had their carious lesions removed and cavity preparation was done with the Vector system (Duerr Dental, Bietigheim-Bissingen, Germany). Cavity preparation was done according to manufacturer's instructions with a special diamond bur with 1.8 mm of diameter. During cavity preparation, a chronometer was used to time each procedure. 

Crowns were separated from the roots with a separator. The prepared cavities were kept in ultrasounded solution for 30 minutes to eliminate dust and other tooth particules. According to electron microscopy instructions, specimens were coated with gold (150 seconds) with the Snonputter technique (Polaron Sputter Coater) for electron microscopy images. 

Prepared cavities were evaluated under a scanning electron microscopy (Jeol JSM-5600, SEM) and dentine and enamel surface roughness and efficiency of carious tissue removal were studied.

## 3. Results

Cavity preparation times with the Vector system and the aerotor are shown in Table 2. The time required for cavity preparation using the Vector system was much longer than the aerotor treatment. 

## 4. SEM Evaluation

### 4.1. Cavity Floor

**Figure 1:**
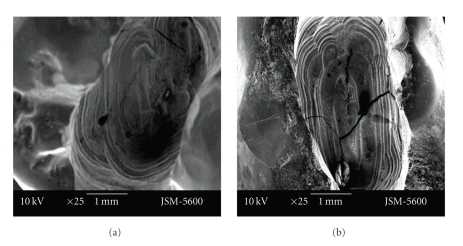
Representative photographs of cross cut section of permanent (a)/primary (b) tooth cavity, prepared by aerotor (magnification ×25).

**Figure 2:**
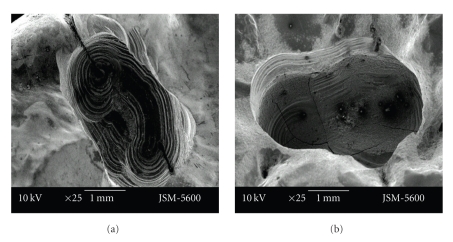
Representative photographs of cross cut section of permanent (a)/primary (b) tooth cavity, prepared by vector system (magnification ×25).

There were bigger and deeper cracks and fractures on the floor of the cavities prepared with the aerotor in comparison to the vector system. In cavities which were prepared with the vector system, cracks were smaller and less deeper. Cavity bordes which were prepared with the Vector system were also as smoother (Figures 1(a)-1(b), Figures 2(a)-2(b)). 

### 4.2. Dentine

In the dentine surface, the orifice of dentinal tubules was seen almost plugged. Typically, these surfaces have previously been described as scaly or flaky, or as an irregular surface.

**Figure 3:**
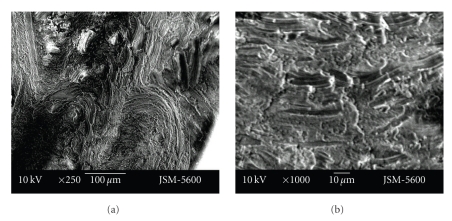
Representative photographs of cross cut section of permanent tooth dentine surface, prepared by aerotor (magnification ×250 and ×1000).

**Figure 4:**
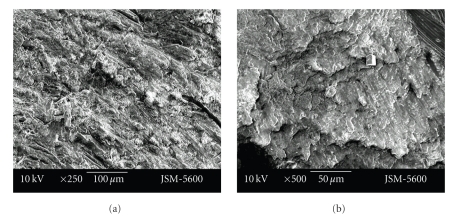
Representative photographs of cross cut section of permanent tooth dentine surface, prepared by vector system (magnification ×250 and ×500).

Smear layer was less evident at the cavities prepared with the vector system. While in cavities prepared with the Vector system open tubule orifices could be observed, at cavities prepared with the aerotor it was observed that most of the tubule orifices were obstructed with a smear layer. Also, the dentin surface of the cavity floor was smoother in cavities prepared with the Vector system in comparison to the aerotor (Figures 3(a)-3(b), Figures 4(a)-4(b)). 

**Figure 5:**
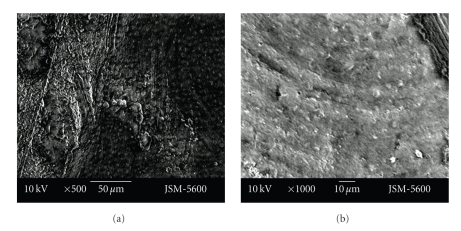
Representative photographs of cross cut section of primary tooth dentine surface, prepared by aerotor (magnification ×500 and ×1000).

**Figure 6:**
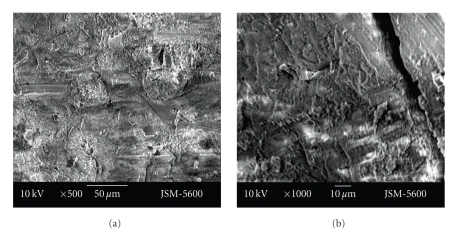
Representative photographs of cross cut section of primary tooth dentine surface, prepared by vector system (magnification ×250 and ×500).

Although cavity surfaces seem relatively smooth at ×250 magnification, rough cavity surfaces with irregular particules were seen at the higher magnifications (×500, ×1000). More smear layer was seen in cavities prepared with the Vector system than those prepared with the aerator. At vector system cavities, indented structure of dentin tubuluses was observed much more clearly. Particules were seen as to be nailed to the smooth dentin surface in the cavities prepared with the Vector system. Tubule entries were observed much clearer with ×1000 enlargement. But tubules were observed as obstructed with debris, poor quality, and pointed surface view (Figures 5(a)-5(b), Figures 6(a)-6(b)). 

### 4.3. Enamel

**Figure 7:**
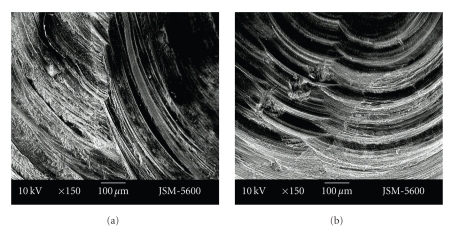
Representative photographs of cross cut section of permanent (a)/primary (b) tooth enamel surface, prepared by aerotor (magnification ×150).

**Figure 8:**
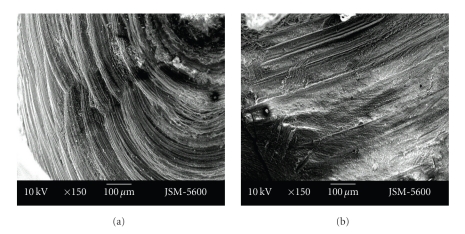
Representative photographs of cross cut section of permanent (a)/primary (b) tooth enamel surface, prepared by vector system (magnification ×150).

Enamel surface morphology was determined similarly in both cavity preparation systems. Enamel lines were streamlined in both the aerotor and vector system (Figures 7(a)-7(b), Figures 8(a)-8(b)). 

## 5. Discussion

This pilot study determined that it is possible to remove carious tissue and perform cavity preparation with the Vector system. According to this preliminary evaluation of surface quality, a cavity prepared with the Vector treatment system is slicker, and the enamel-dentine line is more regular than that prepared with an aerator. However, the Vector system requires a longer treatment time.

The necessity of using the mechanical nonrotatory instruments has emerged to eliminate the negative effects of the conventional methods. One of these is the removal of carious tissue by chemomechanical methods. Chemomechanical caries removal involves the selective removal of soft carious dentin without the painful removal of sound dentin. The Carisolv system that is a popular chemomechanical system for caries removal consists of a gel with amino acids and sodium hypochlorite, and special hand instruments. One of the major advantages is the increased patient compliance to this technique of removing carious dentin compared to drills. In addition, unwanted removal of sound dentin is avoided and the need for local anesthesia is less. However, most of the studies reported that this method prolonged treatment time when compared with rotatory instruments [4, 8]. 

It should be noted that there is as of yet no evidence in the current literature of using the Vector system for minimal invasive cavity preparation (in spite of manufacturer recommendations), and hence the initiation of this pilot study. Therefore there has been no means of comparing these results with others. But after applying this method, it is evident that there could be advantages such as high level of patient acceptance because of reduced pain response, reduced risk of injury due to other tissue sound pulp protection (in deep lesions), high touch sensitivity and minimal use of water cooling (aerosols eliminated and infection minimized). There are some limitations of the method such as reduced visualization due to suspended particles (but there is a possibility of additional flushing of the preparation site), reduced removal rate compared with rotating burs, and the method is not suitable for the removal of extensive soft dentine caries. However, on the basis of the findings reported here, further research in a longer term prospective study comparing the Vector system with a conventional approach using high speed burs, local analgesia, and a rubber dam is now needed [6, 8]. The time required for preparation with the Vector system was significantly longer (9.5 minutes for CS and 16.8 minutes for CM) than when using conventional method (CS 3.9 minutes, CM 5.5 minutes *t*-3.91; *P* < .0002) [6]. On the other hand, all results consistently showed a definite advantage of the rotatory instrument approach with respect to time. 

Pain perception by Hochmans scale showed that 54.8% of individuals treated by the use of the Vector system did not experience any pain as opposed to 29.1% using the conventional method. Children felt the conventional method to be more painful than the Vector system [6]. Rotatory instruments have some disadvantages, such as the nonselective removal of hard tissue, unpleasentness to the patients, necessity of local anesthesia, and potential adverse effects to the pulp due to heat and pressure [3, 9]. It is suggested that the Vector systems have higher patient acceptance, and reduced pain response (sensitive children, anesthetic is often not needed) [8, 10].

The cavities prepared by a diamond bur in a conventional high-speed drill showed a box shaped configuration, with sharp cavo-surface edges and well-defined geometric internal angles, flat floor and wall cavity, with smear layer covering enamel and dentin surface in a typical morphological pattern as observed in Freitas et al.'s studies [3]. In contrast to diamond instruments, the energy is transmitted indirectly via the silicon carbide particles carried in the water with the Vector system. Preparation with the Vector system is therefore carried out without exposure to high temperatures and is extremely gentle on the pulp. Loosening of the enamel prisms is avoided, which results in optimum marginal qualities for subsequent restorations, and with a lesser smear layer than cavity preparation with diamond bur.

## 6. Conclusion

The present study determined that it is possible to remove carious tissue and perform cavity preparation with the Vector system. According to both primary and permanent findings, evaluating surface quality, a cavity prepared with the Vector treatment system has a cavity floor that is slicker and a more regular enamel-dentin line than a cavity prepared with an aerator. While being soundless, nonvibrating, and not using local anesthesia are advantages for clinical practice, a longer treatment time is a negative point, especially for young patients.

## Figures and Tables

**Table 1:** Classification of techniques available for carious dentine excavation.

Category	Technique
Mechanical, rotatory	Handpieces and burs
Mechanical, non rotatory	Hand excavators, air-abrasion, air-polishing, ultrasonics, sono-abrasion
Chemo-mechanical	Caridex, Carisolv, enzymes
Photo-ablation	Lasers

**Table 2:** Cavity preparation time for Aerotor and Vector system.

	Average time
*Primary teeth* prepared with *Vector system *	5.7 minutes
*Permanent teeth* prepared with *Vector system *	7.7 minutes
*Primary teeth* prepared with *Aerotor *	1.5 minutes
*Permanent teeth* prepared with *Aerotor *	1.66 minutes
